# Atomoxetine might be more effective in improving sluggish cognitive tempo symptoms after switching from methylphenidate: A case report

**DOI:** 10.1002/ccr3.3592

**Published:** 2020-11-29

**Authors:** Akın Tahıllıoğlu, Eyüp Sabri Ercan

**Affiliations:** ^1^ Department of Child and Adolescent Psychiatry Ege University Faculty of Medicine Izmir Turkey

**Keywords:** atomoxetine, attention‐deficit/ hyperactivity disorder, methylphenidate, sluggish cognitive tempo, treatment

## Abstract

Although there is no proven evidence regarding pharmacotherapy of Sluggish Cognitive Tempo (SCT), we experienced atomoxetine had more effects in decreasing SCT symptoms after switching from methylphenidate in a case with SCT and subthreshold ADHD.

## Graphical abstract

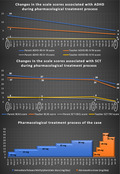

## 1 INTRODUCTION

We report the differential diagnostic procedure and pharmacological treatment process of a case with Sluggish Cognitive Tempo (SCT) and subthreshold ADHD. The case had much more benefit from atomoxetine in terms of SCT symptoms after switching from methylphenidate.

Sluggish Cognitive Tempo (SCT) is a disorder characterized by a range of symptoms such as mental slowness, daydreams, lack of energy, and staring with empty eyes.^1^ Although SCT and attention‐deficit/ hyperactivity disorder (ADHD) are often presented together, increasing current evidence supports that SCT is a separate and independent diagnostic entity differentiating from ADHD. Many studies highlight the overlap between SCT and ADHD. A study found that 44%‐54% of patients with SCT had ADHD diagnosis, 27%‐35% of patients with ADHD had SCT characteristics, and 28%‐46% of children with SCT present an independent phenotype not having ADHD or depression.^2^ Although SCT is more commonly seen with the inattentive presentation of ADHD (ADHD‐IN), these two structures should be evaluated as “closely related with each other, but distinct from each other.” For instance, “daydreaming” and “sluggishness/sleepy appearance” are crucial characteristics to distinguish SCT from ADHD‐IN.^3^ Furthermore, problems on sustaining attention seem more specific to ADHD‐IN, while problems on engaging attention more likely seem to be more specific to SCT.

Evidence on possible treatment modalities of SCT is very scarce. Limited studies suggest that methylphenidate improves attention problems, although it does not improve core SCT symptoms.^4^ Some studies indicate that methylphenidate has no association with improving SCT symptom load.^5^, ^6^ A current study also points out that sluggish/sleepy symptoms do not respond to methylphenidate, whereas daydreamy symptoms have no association with methylphenidate nonresponse.^7^ A study, in which atomoxetine was used, showed that SCT symptom scores decreased as a result of 16 weeks of treatment of atomoxetine, and academic improvement was observed.^5^ This study is important because it was the first study demonstrating improvements in SCT symptoms with medication. On the other hand, another double‐blind placebo‐controlled study suggested that atomoxetine provides control over ADHD symptoms but has minimal effect on SCT symptoms.^8^

When existing data are scrutinized, the lack of studies on SCT treatment is noticeable. In addition, there is no case report on the differential diagnostic process and psychopharmacological treatment process in individuals with SCT. Exploring how the standard ADHD treatment protocol affects SCT symptoms in a case with subthreshold ADHD, and high SCT symptoms, is very crucial for clinicians in terms of shaping their treatment approaches about the patients with SCT. Hence, we aimed to investigate the differential diagnostic procedure of a case with SCT and subthreshold ADHD with long‐term follow‐up. We also aimed to determine whether there are improvements in SCT symptoms and to what extent there has been improvement along with the pharmacological treatment process.

## 2 CASE HISTORY

The 5‐year‐and 6‐month‐old boy was first referred to our outpatient clinic with complaints of being too stagnant compared to their peers, not looking at someone when his name was called, slow‐moving, absent‐mindedness, fear of darkness, and not being able to go to the toilet alone. We learned that a neurological examination by a neurologist had been performed, and no abnormal neurological examination finding had been detected. Besides, no finding in favor of absence epilepsy in the electroencephalography (EEG) examination had been reported. The case was not diagnosed with ADHD and SCT when he was first referred to our clinic. We first had to rule out some neurodevelopmental disorders such as autism spectrum disorder (ASD). In accordance with this, several clinicians observed the manners, behaviors of the patient at different times. He was also observed by a pedagogue in a structured playroom observation. However, no symptom related to ASD was observed. Some inattentive symptoms and SCT symptoms in the case were noticeable, but, the clinical picture was not so clear at that time. During this period, we applied to nonpharmacological interventions including social activities, sport, kindergarten, attention‐enhancing games. However, these interventions did not lead to prominent improvements on SCT symptoms.

After his adaptation to primary school, he learned to read and write on time, and his academic achievement was at the level of the class average. When he was seven years old, he filled the Children Depression Inventory (CDI) ^9^ in order to be examined for depression. He got only 4 points from the CDI (the cutoff point is 19). Clinical psychiatric examinations revealed that there were not enough signs of inattention leading to impairment in daily functionality. He also had no signs of hyperactivity and impulsivity. His teacher reported that he used to move very slowly, look at something for a long time with empty eyes, daydream, but academically did not have problems in his terms of lessons.

**Figure 1:**
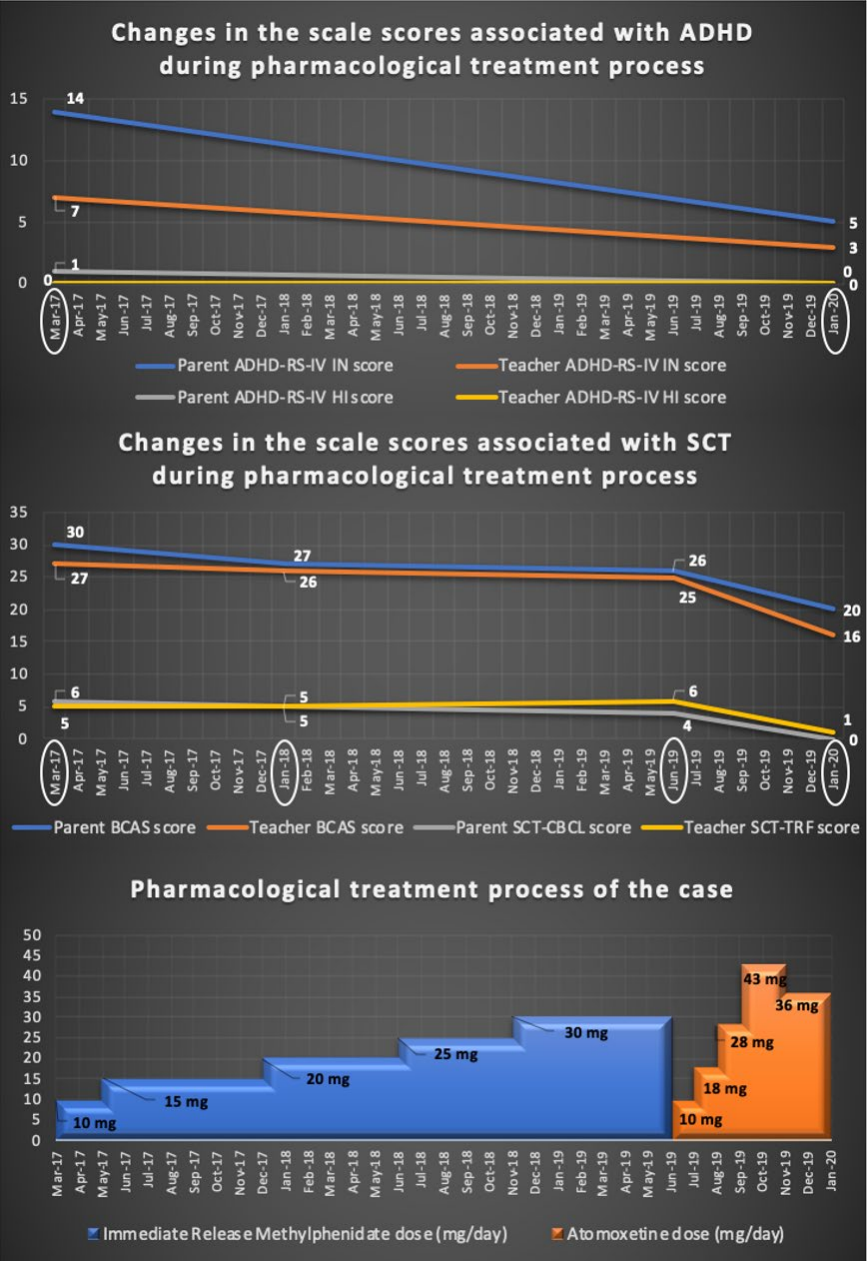
Graphs of the patient's pharmacological treatment history and scores from scales. ADHD‐RS‐IV: DSM‐IV Based Screening and Evaluation Scale for Disruptive Behavior Disorders (IN: Inattention subscale (scoring between 0 and 27 points), HI: Hyperactivity/impulsivity subscale (scoring between 0 and 27 points), BCAS: Barkley Child Attention Survey (scoring between 12 and 48 points), SCT‐CBCL: SCT‐related 4 items of the Child Behavior Check List (scoring between 0 and 8 points), SCT‐TRF: SCT‐related 4 items of Teacher's Report Form (scoring between 0 and 8 points)

When he was 7 years and 4 months old, he got 30 points from the Barkley Child Attention Survey (Barkley SCT Screening Scale—the BCAS) filled by the parents.^3^, ^10^ The BCAS score filled by the teacher was 27. Russell Barkley, who created the BCAS and with whom we had personal communication, stated that 23 points could be determined as a cutoff. He got 6 points from parent‐rated 4 SCT scanning items of Child Behavior Check List (the SCT‐CBCL),^11^, ^12^ and 5 points from teacher‐rated 4 SCT scanning items of Teacher's Report Form (the SCT‐TRF).^12^, ^13^ The thresholds of both the SCT‐CBCL and the SCT‐TRF are 4 points.^14^ A semi‐structured interview, Schedule for Affective Disorders and Schizophrenia for School‐Age Children ‐Present and Lifetime Version (K‐SADS‐PL),^15^, ^16^ was applied to the case and the parents to identify comorbid psychopathologies. According to the K‐SADS‐PL, subthreshold inattentive symptoms not meeting ADHD diagnostic criteria were detected. Inattention scores of parent and teacher‐rated DSM‐IV Based Screening and Evaluation Scale for Disruptive Behavioral Disorders (ADHD Rating Scale – IV, ADHD‐RS‐IV) ^17^, ^18^ were 14 and 7 points, whereas hyperactivity/impulsivity scores were 1 and 0, respectively. Then, the Wechsler Intelligence Scale for Children—Revised Version (WISC‐R) ^19^, ^20^ was applied. It was established that he had normal intelligence capacity with a verbal Intelligence Quotient (IQ) score of 88, a performance IQ score of 128, and a total IQ score of 108. His symbol coding test score was also quite high. After all the evaluations applied to the case, when the case was 7 years and 4 months old, he was considered as having SCT + subthreshold inattentive presentation of ADHD. In order to treat his subthreshold ADHD and SCT symptoms, immediate‐release methylphenidate was started at a dose of 10 mg/d. After two months, he benefited from the drug in terms of moderate attention problems, but the SCT symptoms did not diminish. The dose of methylphenidate was increased to 15 mg/d. Within the next 6 months, SCT symptoms such as daydreaming, staring with empty eyes, and slow‐moving remained at similar severity. Within this particular process, the dose of methylphenidate was increased up to 30 mg/d. During this period, parent‐rated and teacher‐rated BCAS scores were 26 and 25 points, respectively. He got 4 points from the SCT‐CBCL and 6 points from the SCT‐TRF. Although moderate inattentive symptoms decreased, 27 months of methylphenidate use were not sufficiently effective on SCT symptoms. Indeed, teacher and parent reports confirmed this condition. The pharmacotherapy table and the graphs regarding the scores obtained from the scales are shown in Figure 1.

When the patient was 9 years and 7 months old, since he did not benefit enough from methylphenidate in terms of SCT, the medication was switched to atomoxetine. The starting dose of atomoxetine was 10 mg/d, and the dose was increased up to 43 mg/d within 3 months. The dose of atomoxetine was fixed at a dose of 43 mg/d and followed for two months at this dose. The family and the patient reported that he had a headache and dizziness as side effects. For this reason, the dose of atomoxetine was slightly reduced to 36 mg/d. After 2 months of medication at this dose, the headache disappeared, and dizziness decreased significantly. He used atomoxetine for 7 months in total. Clinical examinations and the observation notes obtained from the teacher and the family indicated that there were more significant improvements in SCT symptoms compared to the period when methylphenidate was used. It was determined that moderate inattention problems substantially decreased. There were also noticeable improvements in SCT symptoms such as absent‐mindedness, long reaction time when his name was called, and staring with blank eyes. Parent‐ and teacher‐reported BCAS scores decreased to 20 points and 16 points, respectively. Eventually, the SCT‐CBCL score was 0, and the SCT‐TRF score was 1 point (Figure 1). K‐SADS‐PL was re‐administered approximately 3 years after the first practice. No additional diagnostic comorbidity was detected, except subthreshold inattentive symptoms. The case and his parents were informed of the case study, and written informed consent was obtained from them. This case study was approved by the Ethical Committee of Medical Researches of Ege University (decision no: 20‐2T/7).

## 3 DISCUSSION

### 3.1 Differential diagnostic process in the case with SCT

It is well known that SCT is less associated with externalizing disorders (ie, disruptive behavioral disorders), whereas more associated with internalizing disorders (ie, anxiety, depression) as opposed to ADHD.^21^, ^22^, ^23^ In our case, no evidence was found to suggest the presence of oppositional defiant disorder or conduct disorder. But, especially at the beginning of clinical follow‐up, the presence of anxiety symptoms is consistent with the literature and is expected. However, there were no additional signs of anxiety disorder to diagnose the patient. The case also had not enough evidence to be diagnosed with depression. He was moving slowly and had low energy. These findings may perhaps be confounded with the “anergia” symptom of depression. Even if it was accepted as anergia, the presence of “anergia” alone would not adequately explain the depression in the case.

It was also noted that the patient did not immediately look when his name was called, especially around 6 years of age. Hence, a possible ASD diagnosis was investigated. However, the lack of any impairment in language, social communication, and empathy kept us away from considering ASD. A study found SCT symptoms to be significantly higher in adolescent cases with ASD.^24^ An up‐to‐date study in young adults with ASD found that one‐third of these cases had high levels of SCT symptoms.^25^ The coexistence of these two psychopathologies may be associated with a possible similarity or overlap between SCT and ASD symptoms. Since the "processing speed slowness," which is observed in SCT, can also be observed frequently in individuals with intellectual disability, it is important to distinguish these two different structures. For this purpose, we applied WISC‐R and found that the patient had a normal estimated IQ score and had no mental insufficiency. It is interesting to note that, although the performance IQ and symbol coding test scores of the patient were quite high, the case also demonstrated severe SCT symptoms. Therefore, clinicians should be aware that there might be possible discrepancy between the test performance and daily functioning in the patients having SCT.

### 3.2 Treatment process in the case with SCT

Although our patient did not fulfill the diagnostic criteria of ADHD accurately, we did not want to allow his subthreshold ADHD symptoms and SCT symptoms to remain untreated. An open‐label clinical trial suggests that subthreshold ADHD symptoms responded positively to six weeks of atomoxetine treatment in adults demonstrating atypical manifestations or insufficient symptoms of ADHD (ADHD‐Not Otherwise Specified).^26^ During 27 months of methylphenidate use, the case demonstrated a small reduction in both parent‐ and teacher‐reported SCT scores. An up‐to‐date study suggested there were improvements in the SCT total and SCT‐Daydream scores both at home and at school after the use of methylphenidate, and SCT‐Sluggish scores were found to have improvements only at school (not at home). The study also claimed that the presence of SCT symptoms in children with ADHD had negative effects on methylphenidate treatment in the school area.^27^ Another study pointed out that SCT‐sluggish/sleepy appearance symptoms did not respond to methylphenidate, whereas SCT‐daydreaming symptoms were not associated with methylphenidate nonresponse.^7^ Similar to this study, our case had no significant improvement in “sluggish” symptoms such as slow movement, absent‐mindedness, and sleepy appearance, especially when medicated with methylphenidate. However, unlike these two studies, we did not detect significant improvements in daydreaming symptoms despite methylphenidate. Given both the existing studies and our case report, it might be assumed that “sluggish” symptoms may be more difficult to be treated with methylphenidate. But, the improving effect of methylphenidate on “daydreamy” symptoms should be clarified in future studies, since it seems controversial across the literature and our case.

Wietecha's study is the first study showing improvement in SCT symptoms with atomoxetine. In this double‐blind placebo‐controlled study, three groups diagnosed with "ADHD + Dyslexia," "ADHD only," and "Dyslexia only" were initiated atomoxetine. After 16 weeks of treatment, significant reductions in both ADHD and SCT symptoms were detected in all three groups. A positive correlation was also determined between ADHD and SCT symptoms in terms of improvements in these symptoms with atomoxetine.^5^ In line with this study, after switching the medication and using atomoxetine for 7 months, our patient showed higher rates of decline in SCT scores. Compared to methylphenidate, atomoxetine has more noradrenergic effects in the brain. Hence, atomoxetine might be a more goal‐directed treatment option to improve SCT symptoms which are basically thought to be a symptom cluster of vigilance. Based on this approach, it can also be assumed that modafinil, which leads to vigilance, might be beneficial in improving SCT symptoms.

Our case report has some strengths and limitations. First of all, this case report provided the SCT‐related treatment responses to both methylphenidate and atomoxetine in the same case. Therefore, the implications from this case might help clinicians to better analyze and develop more appropriate treatment strategies regarding SCT. In addition, the absence of other comorbidities including threshold ADHD made treatment response more “SCT‐sensitive.” Furthermore, in order to detect and confirm SCT, both the parents and the teacher were asked to fill the scales related to SCT periodically. In this way, information related to SCT symptoms was obtained from multiple informants. These scales also monitored the pharmacotherapy process, making it easier to find out the results of treatment effectiveness.

As for the limitations, the results cannot be generalized for all cases with SCT since the findings are valid for one case alone. The findings should be replicated, and clinical trials with a higher number of cases are needed.

In conclusion, both methylphenidate and atomoxetine, which are commonly used for ADHD, were used for sufficient time in a case with SCT and subthreshold ADHD. Both methylphenidate and atomoxetine improved moderate inattentive symptoms. Although there were no high response rates to SCT in both drugs, SCT symptoms have decreased much more and faster with the use of atomoxetine. These results should be replicated, and randomized controlled trials are needed with more patients.

## CONFLICT OF INTEREST

ESE is on the advisory board for Sanofi Turkey. AT declares that there is no conflict of interest.

## AUTHOR CONTRIBUTIONS

AT: contributed to the processes of investigation, validation, formal analysis, original draft preparation, review, and editing. ESE: contributed to the processes of conceptualization, methodology, investigation, validation, review and editing, and supervision.

## ETHICAL CONSIDERATIONS

The case and his parents were informed of the case study, and written informed consent was obtained from them. This case report was approved by the Ethical Committee of Medical Researches of Ege University (decision no: 20‐2T/7).

## Data Availability

We have not shared the data from this study yet.
